# Genetic diversity and spatial distribution of *Burkholderia mallei* by core genome-based multilocus sequence typing analysis

**DOI:** 10.1371/journal.pone.0270499

**Published:** 2022-07-06

**Authors:** Sandra Appelt, Anna-Maria Rohleder, Daniela Jacob, Heiner von Buttlar, Enrico Georgi, Katharina Mueller, Ulrich Wernery, Joerg Kinne, Marina Joseph, Shantymol V. Jose, Holger C. Scholz

**Affiliations:** 1 Centre for Biological Threats and Special Pathogens (ZBS 2), Robert Koch Institute, Berlin, Germany; 2 Bundeswehr Institute of Microbiology, Department Bacteriology and Toxinology, Munich, Germany; 3 Central Veterinary Research Laboratory, Dubai, United Arab Emirates; Tianjin University, CHINA

## Abstract

*Burkholderia mallei* is the etiological agent of glanders, a highly contagious and often fatal disease in equids. Due to the high genetic clonality of *B*. *mallei*, high-resolution typing assays are necessary to differentiate between individual strains. Here we report on the development and validation of a robust and reproducible core genome-based Multi Locus Sequence Typing Assay (cgMLST) for *B*. *mallei*, which is based on 3328 gene targets and enables high-resolution typing at the strain level. The assay was validated using a set of 120 *B*. *mallei* genomes from public databases and 23 newly sequenced outbreak strains from in-house strain collections. In this cgMLST analysis, strains from different geographic regions were clearly distinguished by at least 70 allele differences, allowing spatial clustering while closely related and epidemiologically related strains were separated by only zero to three alleles. Neither the different sequencing technologies nor the assembly strategies had an influence on the cgMLST results. The developed cgMLST is highly robust, reproducible and can be used for outbreak investigations, source tracking and molecular characterization of new *B*. *mallei* isolates.

## Introduction

*Burkholderia mallei* is a small rod-shaped, Gram-negative bacterium that causes glanders, a notifiable disease in animals and humans [1]. Glanders is extremely contagious among *Equidae*, especially for horses and donkeys [2, 3]. In many cases, infected animals need to be euthanized following governmental regulations on the eradication of epizootic diseases. Although glanders has been extirpated from Western Europe, Northern America, and Australia, it has recently gained the status of a re-emerging disease due to constantly increasing numbers of outbreaks in Africa, Asia, and South America [4–6].

Humans are considered incidental hosts, with no cases reported in recent years, with the exception of one laboratory accident in the United States [7] and a suspected case of glanders in an Brazilian child [8]. In the past, the reported human glanders cases mainly concerned veterinarians, horsemen or abattoir workers, and only rarely representatives of other professions [9].

*Burkholderia mallei* can be described as a recently evolved, host-adapted clone (lineage) derived from *Burkholderia pseudomallei*, the causative agent of melioidosis [10]. During adaption to the host, *B*. *mallei* underwent a significant genome reduction resulting in a set of two chromosomes with average sizes of about 3.5 and 2.3 Mbp compared to average chromosome sizes of 4.1 and 3.2 Mbp for *B*. *pseudomallei*. In *B*. *mallei*, essential genes are mainly encoded on the larger primary chromosome, while biological niche-specific genes are bound to the smaller secondary chromosome [11, 12]. *B*. *mallei* is known for its remarkable genome plasticity, mainly caused by insertion element-driven large-scale genetic re-arrangements, suggesting an intermediate evolutionary stage [11]. On the other hand, and in contrast to *B*. *pseudomallei*, *B*. *mallei* populations are also known to be highly clonal and rarely show variations in coding gene sequences. As a consequence, the classical MLST-7 assay is useful for investigating the genetic population structure of *B*. *pseudomallei*, but fails to discriminate among *B*. *mallei* strains [13–15]. Based on this difficulty to delineate individual *B*. *mallei* strains, high resolution typing assays are required [13]. In previous studies both Multi Locus Variable Number of Tandem Repeats analysis (MLVA) and Single Nucleotide Polymorphism (SNP) analysis were proved to be suitable for high-resolution typing of *B*. *mallei* strains [13–19]. While SNP genotyping ensures robust and reproducible analyses, VNTR profiles generated by different laboratories may vary significantly [20]. Furthermore, VNTR-regions may change rapidly upon laboratory passage but also during short term acute infection and, thus, produce different results which makes it difficult to interpret epidemiological linkages correctly [21]. Consequently, when it comes to the interpretation of VNTR typing results, exact strain identification and sample attribution during source tracking or outbreak investigations remain uncertain, unless a hierarchical typing approach consisting of SNP- or cgMLST analysis followed by MLVA is applied. Like SNP analysis, cgMLST offers high genetic resolution but also an excellent reproducibility combined with a proven robustness as shown for many other bacterial genera, like for example *Brucella*, *Yersinia*, *Klebsiella*, *Enterobacter* and *Enterococcus* [22–25]. Additionally, cgMLST has the advantage over varying SNP assays of being readily and consistently applied in different laboratories as it uses a consistent set of well-defined conserved loci and allele designations. Whereas a cgMLST assay has been published for *B*. *pseudomallei* recently [26], for *B*. *mallei* no cgMLST assay was available.

In this study, we consequently developed and validated a core genome based MLST (cgMLST) scheme using the SeqSphere+ software, which is based on 3328 gene targets and enables exact typing of *B*. *mallei* at the strain level. Genetic relationships and geographical attributions were performed by applying this assay to a dataset of 120 *B*. *mallei* genomes. By including twenty-three newly sequenced strains from two different outbreaks of glanders, it was also possible to determine the natural diversification of *B*. *mallei* in a short-term scenario in its natural host. In addition, cluster analysis was used to establish provisional cutoffs for strain demarcation, which make it possible to identify identical *B*. *mallei* strains and to distinguish between epidemically unrelated strains. A number of 5 *B*. *mallei* genomes with incorrect strain designation could be identified which were deposited in the NCBI bacterial genome database.

## Material and methods

### Bacterial strains of the in-house collection and whole genome sequencing

To complement the 83 *B*. *mallei* genome sequences available in the NCBI genome database and for assay validation purposes, 37 *B*. *mallei* strains from German strain collections (36 from the Bundeswehr Institute of Microbiology and one from the Robert Koch-Institute) were included in the analyses (**Table 1**). These 37 *B*. *mallei* strains include: twenty-three strains derived from previously described outbreaks of glanders in race horses and a dromedary in the United Arab Emirates and Bahrain (**Table 1**), six *B*. *mallei* reference strains received from the National Collection of Type Cultures (NCTC), the *B*. *mallei* type strain ATCC 23344T (L3_0558), *B*. *mallei* Mukteswar (L3_0580) and two *B*. *mallei* Zagreb strains (L3_0586, A104-4) plus four strains with unknown origin and history (L3_543, L3_552, L3_554, and L3_572).

**Table 1:** Information on the sequenced *B*. *mallei* genomes included in this analysis.

ID*	Aliases	Collection year	Country of isolation	Host	NCBI Short Read Archive (SRA) Accession IDs
L3_0543*	strain 32	1972	unknown	unknown	SAMN19373946
L3_0552	Not attributed	unknown	unknown	unknown	SAMN19373947
L3_0554	Not attributed	unknown	unknown	unknown	SAMN19373948
L3_0558	ATCC 23344T	1944	China	human	SAMN19373949
L3_0572	Not attributed	unknown	unknown	unknown	SAMN19373950
L3_0580*	Mukteswar	1996	India	horse	SAMN19373951
L3_0586*	Zagreb	1996	Yugoslavia	unknown	SAMN19373952
L3_0762	NCTC 3709; 106	1932	India	horse	SAMN19373953
L3_0764	NCTC_120 (Lister strain), strain A	1920	United Kingdom	unknown	SAMN19373954
L3_0765	NCTC 10260, strain 11	1949	Turkey	unknown	SAMN19373955
L3_0766	NCTC 10247, strain 12	1960	Turkey	unknown	SAMN19373956
L3_0767	NCTC 10245, China5, ATCC 10399	1972	China	horse	SAMN19373957
L3_0768	ATCC 15310, NCTC 10230, IVAN	1961	Hungary	horse	SAMN19373958
L3_2399*	UAE 7, 6SK2, Al Ain Dubai, ANEEF	2004	United Arab Emirates	horse (Aneef)	SAMN19373959
L3_2955*	Dubai 3, No.6 SK3 v. D2115/04	2004	United Arab Emirates	horse (Aneef)	SAMN19373960
L3_2956*	Dubai 4, No.9 SK1 v. D2115/04	2004	United Arab Emirates	horse (Aneef)	SAMN19373961
L3_2958*	Dubai 6, No.1 v. D2220/04, Guinea pig	2004	United Arab Emirates	horse (Aneef)	SAMN19373962
L3_2959*	Dubai 7, No.2 v. D2220/04, Guinea pig	2004	United Arab Emirates	horse (Aneef)	SAMN19373963
L3_2960*	Dubai 8, No.1 v. D2267/04	2004	United Arab Emirates	horse (Aneef)	SAMN19373964
L3_2962*	Dubai 10, No.1 v. D2268.1/04	2004	United Arab Emirates	horse (Aneef)	SAMN19373965
L3_2966*	Dubai 14, No.1 v. D2567/04	2004	United Arab Emirates	horse	SAMN19373966
L3_2967*	Dubai 15, No.2 v. D2567/04	2004	United Arab Emirates	horse	SAMN19373967
L3_2968*	Dubai 16, No.1 v. D2700/04, Guinea pig	2004	United Arab Emirates	horse	SAMN19373968
L3_2969*	Dubai 17, No.2 v. D2700/04, Guinea pig	2004	United Arab Emirates	horse	SAMN19373969
L3_3269*	SK3 v. D1113.4/10	2010	Bahrain	horse	SAMN19373970
L3_3270*	SK 9 v. D1112.8/10	2010	Bahrain	horse	SAMN19373971
L3_3271	SK2 v. D1282/10 (nostril)	2010	Bahrain	dromedary	SAMN19373972
L3_3272*	SK v. D1257.3/10	2010	Bahrain	horse	SAMN19373973
L3_3273	SK2 v. D1282/10 (blood)	2010	Bahrain	dromedary	SAMN19373974
L3_3314*	Dubai 2, No.6 SK2 v. D2115/04	2004	United Arab Emirates	horse (Aneef)	SAMN19373975
L3_3315*	Dubai 1, No.5 SK2 v. D2115/04	2004	United Arab Emirates	horse (Aneef)	SAMN19373976
L3_3316*	D2231/10	2010	Bahrain	horse	SAMN19373977
L3_3317*	D274/11	2011	Bahrain	horse	SAMN19373978
L3_3318*	D400/11	2011	Bahrain	horse	SAMN19373979
L3_3319*	D401/11	2011	Bahrain	horse	SAMN19373980
L3_3320*	D403/11	2011	Bahrain	horse	SAMN19373981
A104-4	Zagreb	unassigned	Yugoslavia	horse	SAMN21877244

* MLVA/SNP: Scholz, Pearson (14); L3: Culture Collection German Federal Armed Forces, A: Culture Collection Robert Koch Institute

For DNA extraction, *B*. *mallei* isolates of the in-house collections were grown on Columbia Blood agar plates at 37°C and harvested after 48 hours. The Qiagen Genomic Tip kit (Hilden, Germany), was used for extraction of genomic DNA following the suppliers’ instructions. Library preparation, and 250 bp Illumina paired end sequencing (MiSeq), was done by GATC Biotech AG (Konstanz, Germany). Raw reads were supplied as fastq files. For *B*. *mallei* strain Zagreb (A104-4), genomic DNA was extracted with the QIAGEN DNeasy Blood and Tissue Kit (Qiagen, Hilden, Germany). The NextEra XT DNA Sample Preparation Kit (Illumina, San Diego, CA, USA) was used for preparing the library and sequencing in paired-end mode was performed on the MiSeq instrument (Illumina, San Diego, CA, USA).

### Genome assembly

Raw Illumina reads were de-novo assembled using the SeqSphere+ software pipeline (v. 7.2.3, Ridom GmbH, Münster, Germany), SKESA [27] for read assembly. Prior to assembly, raw reads were quality controlled using FastQC [28] and down-sampled to a coverage of 180.

To investigate the impact of different genome assembly strategies, the sequencing reads obtained for two strains (L3_543, and L3_552) were assembled with Velvet [29] and SPADES [30] included in SeqSphere+ and additionally with the CLC Genomics Workbench 11.0 Software (Qiagen, Hilden, Germany). Sequencing reads generated for the sample L3_543 were assembled with and without FastQC.

All genome sequences have been uploaded to the NCBI Short Read Archive (SRA: www.ncbi.nlm.nih.gov/sra). The BioProject submission IDs are PRJNA733297 and PRJNA766820, individual BioSample IDs are provided in the **Table 1**.

### cgMLST target scheme definition

The public available, finished genome of *B*. *mallei* type strain ATCC 23344T (NCBI Accession: NC_006348.1, NC_006349.2) was selected as ‘seed genome’. To determine the cgMLST gene set, a genome-wide gene-by-gene comparison using the cgMLST Target Definer (v. 1.4) function of the SeqSphere+ software (v.7.2.3, Ridom GmbH, Münster, Germany) was performed. The applied default parameters served to exclude certain genes of the ‘seed genome’ from the cgMLST scheme and comprised the following filters: a minimum length filter that discards all genes shorter than 50 bp, a start codon filter that discards all genes that contain no start codon at the beginning of the gene, a stop codon filter that discards all genes that contain no stop codon or more than one stop codon or if the stop codon is not at the end of the gene, a homologous gene filter that discards all genes with fragments that occur in multiple copies within a genome (with identity of 90% and more than 100-bp overlaps), and a gene overlap filter that discards the shorter gene from the cgMLST scheme if the two genes affected overlap by >4 bp.

The remaining genes were included in a pairwise comparison using BLAST (v.2.2.12, parameters as follows: word size, 11; mismatch penalty, −1; match reward, 1; gap open costs, 5; gap extension costs, 2), to the query chromosomes of selected *B*. *mallei* strains. The selected query genomes are publicly available and comprise 13 genomes from *B*. *mallei* strains out of distinct geographical areas with different molecular profiles to cover the entire genetic diversity of *B*. *mallei* (**S1 Table**) [14, 15, 17].

The final cgMLST scheme was formed by including all genes of the reference genome that were common in all query genomes (**S2 Table**) with a sequence identity of ≥90% and 100% of overlap. Also, all genes having no start or stop codon in one of the query genomes, as well as genes that had internal stop codons in more than 20% of the query genomes, were discarded. The final cgMLST scheme consisted of 3328 target genes (55,2% of the reference genome of ATCC 23344T). A template of the cgMLST assay is provided as **S3 Table** and will be made accessible to the public on www.cgmlst.org.

### Setting up the genomic database

The final genome database consisted of 120 *B*. *mallei* genomes: 83 genomes from the NCBI genome database [31] (assessed 1^st^ of February 2020) and 37 newly sequenced *B*. *mallei* strains from the in-house strain collections described above.

### cgMLST-based analysis

To validate the cgMLST scheme, 120 *B*. *mallei* genomes included in the database were analyzed. The genotypic resolution of the assay was monitored by analyzing, genomes from bacterial derivates originating from type strain ATCC 23344T and NCTC reference strains from different culture collections. A broad geographical distribution was achieved by including genomes of *B*. *mallei* strains from different parts of the world: Brazil, Bahrain, China, France, Hungary, India, Iran, Pakistan, Russia, Turkey, United Arab Emirates, United Kingdom, United States of America, and Yugoslavia (**S2 Table**).

In addition, the database comprised genomes constructed from reads generated by different Next-Generation Sequencing platforms (Illumina, PacBio versus 454 Life Science vs PacBio alone (Illumina, PacBio, 454: *B*. *mallei* FMH_23344T; PacBio: *B*. *mallei* FMH (NCBI Accession numbers are provided in **S2 Table**). Also, included were genomes already analyzed by MLVA and SNP [14] (**Table 1**) to test for the repeatability of the typing.

The Pearson chi-squared test with Yates’s correction was applied to test the possibility that identical *B*. *mallei* show zero to three allelic differences among each other [32]. The Yates (1934) correction was applied to prevent overestimation of statistical significance in the small dataset. To run the statistical computing, the free software R version 4.0.4 was used [33]. The outbreak strains from Bahrain and Dubai were excluded from this analysis as well as four isolates: FDAARGOS_585, FDAARGOS_588 to 590 that were not included in the cgMLST analysis due to missing values.

The incorrect designation of strains deposed in public data bases was neglected. The dataset was divided in two groups. Included in group 1 were individual strains and their derivates (including ATCC 23344T and NCTC derivates). Included in group 2 were unique strains with no identified derivates. Group 1 comprised 60 strains, 51 strains showed zero to three allelic differences among each other. Group 2 comprised 41 strains of which 8 showed one to three differences to other strains not designated as identical.

### Phylogenetic reconstructions

For visualizing the distance of single genotypes, a Minimum Spanning Tree was constructed using the globally optimized implementation of the eBURST algorithm [34]. Missing values were ignored during pairwise comparison and samples with missing values in more than 10% of the columns were discarded during the analysis. A total of four *B*. *mallei* genomes (see above) were sorted out of the analyzed dataset because of missing values. Additional, phylogenetic reconstruction was performed with MEGA11 on core genome SNPs exported from SeqSphere. The evolutionary history was inferred using the Neighbor-Joining method. The bootstrap consensus tree inferred from 500 replicates.

Both phylogenetic trees were compared for similarities and differences regarding the clustering of *B*. *mallei* strains.

## Results

### Overall assay performance

The developed cgMLST assay comprises a total of 3328 target genes (55.2% of the total seed genome) that match the stringent parameters described in the Materials and Methods section. The accessory genome comprises further 1302 gene targets, which could optionally be used in a hierarchical clustering approach in order to achieve a higher genetic resolution, if necessary. Finally, 414 gene targets did not meet the stringent criteria and were discarded.

Of 120 *B*. *mallei* genome sequences, 116 were exactly genotyped with a high score of “good gene targets” in the range of 95 to 100%. The remaining four genomes (FDAARGOS_585, FDAARGOS_588–590) showed a lower percentage of good gene targets (73%), but typing was still possible. A detailed analysis of the failed target genes of the four genome sequences indicated errors that presumably occurred during genome assembly (not shown). In the classic MLST-7 analysis [13], 118 strains belonged to sequence type (ST) 40 and two strains, NCTC 10247 and NCTC 10260, belonged to ST 100 (not shown), confirming the high clonality of *B*. *mallei*. The genetic discriminatory power of the developed cgMLST assay was comparable to the SNP and VNTR assays applied in an earlier study [14] to 24 *B*. *mallei* strains (**Table 1**). In summary, the cgMLST-based phylogenetic reconstruction resulted in identical phylogenetic tree topologies with similar genetic resolutions compared to VNTR and SNP typing [14]. Furthermore, neither the use of different sequencing technologies nor the assembly strategies had any influence on the cgMLST results, which underlines the robustness of the assay.

### Spatial clustering of *B*. *mallei*

**Fig 1:**
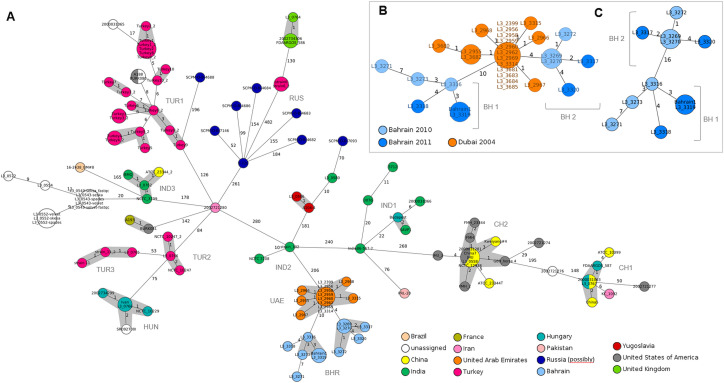
Minimum spanning tree visualizing the clustering of *B*. *mallei* strains. **(A)** Allelic differences and spatial clustering of *B*. *mallei* strains dependent on the reported geographical origin. **(B)** Allelic differences of outbreak strains from Dubai and Bahrain. **(C)** Direct comparison of cgMLST profiles of *B*. *mallei* strains from Bahrain to specify allelic differences within the outbreak cluster. Since the outbreak strains form the United Arab Emirates are excluded in this analysis, the allelic differences vary from the figures shown in (A) and (B). The Bahrain *B*. *mallei* strain formed two distinct clusters: BH1 and BH2. The Minimum Spanning Tree was reconstructed on results provided by the cgMLST analysis. Each sequence type is represented by a single node, nodes are connected if they are single locus variants. The size of nodes refers to the number of genomes with identical locus variants. The color of nodes reflects the reported geographical origin of the *B*. *mallei* strain. For each identified spatial cluster, the internal three-letter code is provided. Each *B*. *mallei* strain is identified by a unique identifier. Numbers along the branches indicate allelic differences. Nodes with less than 3 allelic differences to each other are shaded in grey.

In phylogenetic reconstructions, most of the *B*. *mallei* strains grouped according to their respective geographical origin, which enables a phylogeographical analysis or spatial assignment. A total of eleven different geographical clusters were identified, comprising strains from 7 geographical regions: Bahrain, United Arab Emirates; India, Russia, Hungary, Turkey and China (**Fig 1A and S1 Data**). Two main clusters (CHN1 and CHN2), separated by 177 alleles, were generated for China (**Fig 1A**, detailed in **S1 Fig**). Cluster CHN1 comprised the strain China 5 and its derivatives, cluster CHN2 was built up from genomes of the type strain ATCC 23344T (China 7) and its derivatives. CHN1 also comprised the strain KC 1092 (alias 2002721280) from Iran (see strains with incorrect names below). A number of strains deposited with the United States (USA) as geographic origin can be considered Chinese strains because they are derivatives of the type strain ATCC 23344T (cluster CHN2, **Fig 1A**, detailed in **S1 Fig**). Two other strains with designation of origin USA (Burk080 and 2002721277), grouped with strains from Turkey and China, which also indicates a different origin than the USA. The 28 genome sequences of strains from Turkey formed 3 different geographical clusters, consisting of 20 (TUR1), 3 (TUR2) and 3 (TUR3) genomes (**S2 Fig**), respectively. The largest cluster (TUR1) was formed by ten strains from Turkey (referred to as Turkey 1 to Turkey 10) with only 0 to 8 allele differences between individual strains, which indicates a geographical / epidemiological connection between strains.

The 20 genome sequences of the ten Turkish strains in this cluster were deposited by two different institutions in the NCBI bacterial genomic database and are therefore marked in pairs (Turkey1 / Turkey1_2 to Turkey10 / Turkey10_2). Each related strain pair differed only in 0–1 alleles, despite having been deposited by different institutions and sequenced with different sequencing technologies. TUR 1 also comprised two strains of unknown origin (A188 and 2000031065) and strain BURK080, with USA as given of origin (see above), which indicates a Turkish origin. The two other Turkish clusters (TUR2, TUR3) were each formed from three genomes of the strains NCTC 10247 and strain 11, respectively, which were deposited from different strain collections. Genomes within clusters TUR2 and TUR3 differed in 1 to 2 alleles only, confirming strain identity.

The 11 *B*. *mallei* genomes of Indian origin grouped into a total of three different clusters (IND1, IND2, IND3, detailed illustration **S3 Fig**), consisting of 5, 2, and 3 genomes, respectively. Cluster IND1 comprised five different strains differing in up to 22 alleles and was separated from Cluster IND2 by 240 alleles. IND1 also comprised two clinical isolates (strains 3076 and 3712) recently isolated in 2015 from an infected horse. Clusters IND2 and IND3, separated by 288 alleles, were each composed of genome sequences from aliases of single strains (NCTC 3708 and NCTC 3709, respectively). The high number of differentiating alleles between the clusters IND1-IND3 indicate the presence of at least 3 distinct phylogenetic groups of *B*. *mallei* populations in India. A further single Indian strain (Mukteswar; L3_0758) was separated from Cluster IND2 by 183 alleles (detailed figure) and defined a separate lineage.

Six genome sequences deposited by a Russian institution and labeled with SCPM identifiers but without geographical information formed a genetically more diverse cluster (with 52 to 184 alleles difference), branching of from strain V120, isolated in Russia. It therefore can be assumed that these genome sequences refer to genetically diverse strains from Russia.

The 23 outbreak strains from Dubai and Bahrain were genetically closely related but formed different clusters (for details see **Characterization of outbreak strains)**)

A total of 6 genomes sequences with Hungary as given origin were available. All genomes are derivates of two reference strains NCTC 10229 (alias Budapest) and NCTC 10230 (aliases ATCC 15310, IVAN). Both NCTC strains were isolated in 1961. NCTC 10229 is the original strain, isolated from a bird and NCTC 10230 is a derivative of NCTC 10229 obtained from passage through Guinea pig. In this cgMLST cluster analysis, the 4 NCTC 10229 genomes differed in only one allele. The closest relative was strain NCTC 10247 from Turkey with 75 alleles difference. The three remaining genome sequences, deposited as strains Budapest, FDAARGOS 587, and 2000031063, did not cluster as expected and are described below as Strains not clustering according to their designation.

In summary, like MLVA and SNP analysis, cgMLST allows spatial typing of *B*. *mallei*. In some countries different *B*. *mallei* lineages do exist. However, because of the low number of available genomes in combination with missing metadata, especially of exact geographical origin, final interpretation remains difficult.

### Characterization of outbreak strains

A total of 23 *B*. *mallei* strains from glanders outbreaks in Dubai (nb 13 strains; 2004) and Bahrain (nb 10 strains; 2010/2011) were genome-sequenced and included in this study. Twenty-one of the strains were isolated from horses and two strains were derived from a single infected dromedary (Wernery et al., 2011 [19]) (**Table 1**). From this strain collective, 21 were previously analyzed by MLVA or SNP analysis, respectively (Scholz et al., 2014 [14]) (**Table 1**). As in the previous study, in the current cgMLST analysis, strains from Dubai (UAE) and Bahrain were genetically closely related, but formed different groups (**Fig 1B and 1C**). While Dubai strains were either indistinguishable or differed in only one allele, Bahrain strains were genetically more diverse and formed two different clusters called BH-1 and BH-2, each consisting of six strains (**Fig 1B and 1C**). It is noteworthy that Bahrain strains of the UAE / BH-1 cluster were more closely related to the strains from Dubai (4 allele differences) than to the second Bahrain cluster, BH-2 (10 allele differences, **Fig 1B**). In a direct cgMLST comparison, Bahraini strains of both clusters were clearly separated by 16 alleles (**Fig 1C**), strongly suggesting that the outbreak was caused by different strains. Cluster BH-2 also included the strain Bahrain1, which was deposited in the NCBI database by another research group. This strain was identical to the L3_3319 strain of this study and was therefore probably collected during the same outbreak.

The higher genetic diversity of the Bahraini strains can be explained by the different nature of the two outbreaks. The 2004 Dubai outbreak occurred within a short period of time during quarantine under containment conditions. In contrast, the outbreak in Bahrain lasted more than a year and affected a larger area in the north of Bahrain (Wernery et al., 2011 [19]; Scholz et al., 2014 [14]). In addition, most of the *B*. *mallei* isolates (eight out of 13) from the Dubai outbreak were from different specimens from a single horse named ANNEF (**Table 1**), while the eight horse isolates from the Bahrain outbreak were from eight different horses from different geographic areas. As a result, the strain populations of the two outbreaks exhibited different genetic diversity. Interestingly, the two strains of the same camel (L3_3273 isolated from the nostril and L3_3771, isolated from blood) unexpectedly differed in 7 alleles, which suggests a simultaneous infection with two different *B*. *mallei* strains.

### The *B*. *mallei* type strain ATCC 23344T and derivatives

**Fig 2:**
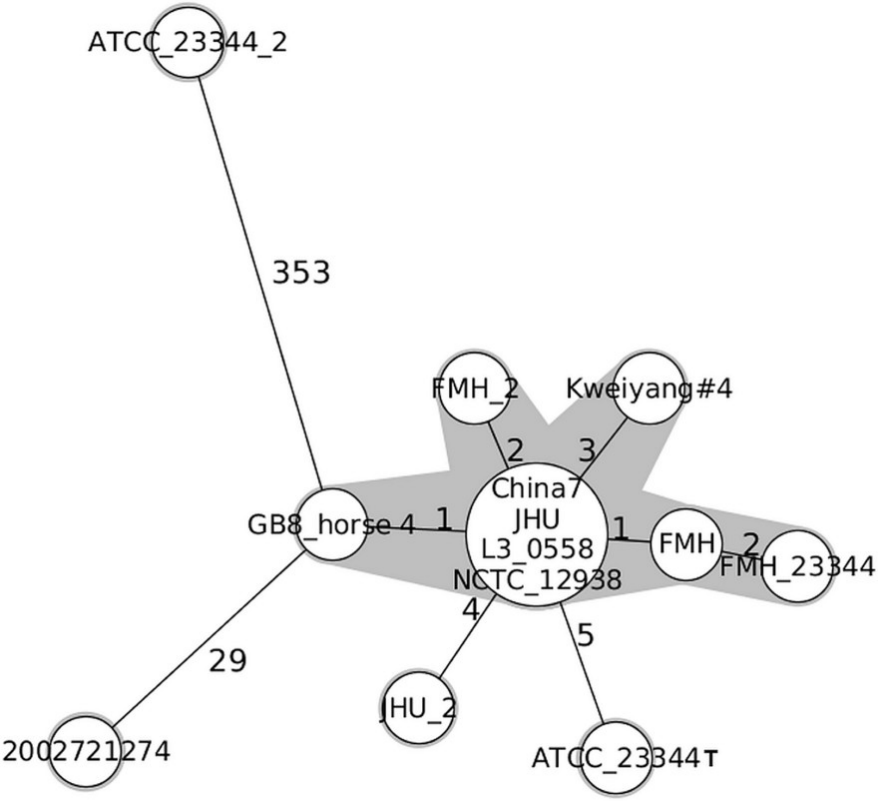
Allelic profiles of ATCC 23344T type strain derivatives. The Minimum Spanning Tree was reconstructed on results provided by the cgMLST analysis. Each sequence type is represented by a single node, nodes are connected if they are single locus variants. The size of nodes refers to the number of genomes with identical locus variants. Number along the branches indicate allelic differences. Nodes with less than 3 allelic differences to each other are shaded in grey. Each *B*. *mallei* strain is identified by a unique identifier.

The type strain ATCC 23344T was originally isolated from the knee fluid of a Chinese soldier in 1949 and has since been used by various laboratories as a reference, but also for animal infection experiments. The genome data set used for the cgMLST analysis comprised two genome sequences deposited as type strains (ATCC_23344T and ATCC 23344T_2) and a further nine genome sequences of its derivatives, submitted under their aliases (e.g. China 7, NCTC_12938) or names from different strain collections (e.g. L3_0558, JHU, FMH). With one exception (ATCC 23344T_2: accession number NZ_CP008704.1, NZ_CP008705.1, see below), all genome sequences deposited as type strain or with their alias names grouped correctly and formed a single cluster with a maximum difference of 5 alleles (**Figs 1A and 2**). Derivatives of the type strain (L3_0558, China7, NCTC 12938, 2000031281) and an isolate (JHU) could not be distinguished when using the cgMLST assay, which confirmed the strain identity. Two genomes of the type strain isolated from an accidentally infected human patient (FMH, FMH 23344T) and another genome of the type strain isolated from an experimentally infected horse (GB8 horse 4) [21] differed in one to two alleles. It is noteworthy that the original type strain ATCC 23344T (NC_006348.1, NC_006349.2 [35], which was sequenced in 2004 using Sanger sequencing technology, was more distantly related and separated from the derivative cluster by five alleles. Taken together, with the exception of a genome sequence with a presumably incorrect affiliation, all genome sequences of the type strain and its derivatives are grouped closely together regardless of the strain history.

### NCTC reference strains from different culture collections

**Fig 3:**
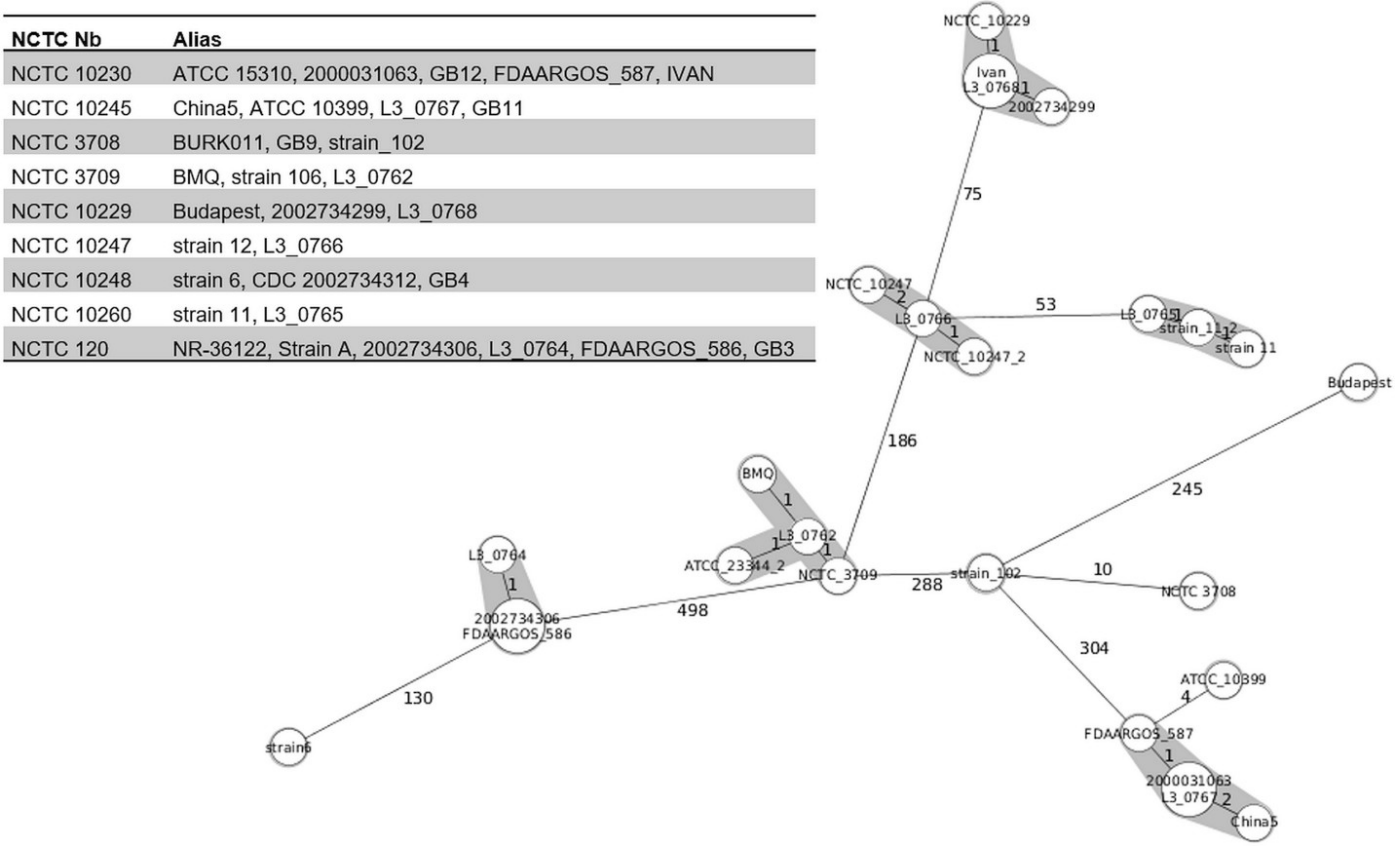
Allelic profiles of NCTC reference strains. The Minimum Spanning Tree was reconstructed on results provided by the cgMLST analysis. Each sequence type is represented by a single node, nodes are connected if they are single locus variants. The size of nodes refers to the number of genomes with identical locus variants. Number along the branches indicate allelic differences. Nodes with less than 3 allelic differences to each other are shaded in grey. Each *B*. *mallei* strain is identified by a unique identifier. Strain ATCC_23344T_2 was potentially misclassified as well as strain Budapest.

The NCTC reference collection included in this study consisted of ten *B*. *mallei* strains (NCTC_120, NCTC_3708, NCTC_3709, NCTC_10229, NCTC_10230, NCTC_10245, NCTC_10247, NCTC_10248, NCTC_10260 and NCTC_12938) from different countries (India, China, Turkey, Hungary, and UK). Only four strains (NCTC_3708, NCTC_3709, NCTC_10229 and NCTC_10247) were available from the NCBI genome database under their respective NCTC numbers, while the other strains were stored with their aliases or individual names of the submitting institution (**S2 Table**). In the cgMLST analysis, all NCTC strains were properly clustered according to their names and geographical origin. Three genome sequences (L3_0746, FDAARGOS 586 and 2002734306) related to the strain NCTC_120, also known as the Lister strain with UK as the given origin. They differed in 0 to 1 allele (**Figs 1A** and **3**). The closest genetic relative (130 alleles difference) was strain 6 with Turkey as the given geographical origin, which makes UK as the geographical origin of the Lister strain unlikely.

Taken together, the genomic sequences of a given NCTC strain and its derivatives, kept in different strain collections or deposited under their aliases, differed in zero to two alleles (**Fig 3**).

### Strains not clustering according to their designation

A total of five *B*. *mallei* genome sequences stored in the NCBI database were identified that did not cluster in accordance with the strain names mentioned (overview **S4 Table and S1 Data**). The genome sequence NZ_CP008704.1, NZ_CP008705.1, designated as type strain ATCC 23344T_2, did not group with the correctly designated type strain (ATTC 23344T: NC_006348.1, NC_006349.2) and all its derivatives (e.g. JHU, FMH, GB 8 Horse). The strain was with only 2 alleles difference most closely related to strain NCTC 3709 from India (**Figs 1A** and **3**). Three other genome sequences that were deposited as strains 2000031063, FDAARGOS_587 and KC_1092 (alias 2002721280) also did not cluster as expected. They were all closely related to the strain China5 and its derivatives (**Fig 1A**). Finally, a strain deposited as Budapest (SAMN04260157) did not group with NCTC_10229 (alias Budapest) and other strains from Hungary. The strain was most closely related (two alleles difference) with the strain SAVP1, an attenuated strain from India (**Fig 1A**).

### Geographical allocation of *B*. *mallei* strains with partial or missing records

For 15 strains metadata were either uncertain, incomplete or missing (**S5 Table**). Using the cgMLST cluster analysis, a possible geographical origin could be determined for 13 strains.

No geographical records were available for four *B*. *mallei* strains of the in-house strain collection (L3_543, L3_552, L3_554, and L3_572). The analysis revealed that, India seems to be the most likely geographic origin, as they branch off from a cluster of strains from India (BMQ, NCTC 3707), with only 20 alleles difference of strain NCTC_3709 (**Fig 1A**). Three strains with identical cgMLST profiles (A188, BURK080, FDAARGOS_585) and the strain 2000031065 with unknown geographical origin were closely related to strains from Turkey with only 5 to 13 allele differences. Therefore, Turkey can be assumed as the origin. The strain FDAARGOS_589 showed only one allele difference from the SAVP1 strain from India and can therefore be regarded as a derivative of this strain. Another strain, 2002721276, was most closely related to strains from China and India, suggesting an Asian origin.

A couple of strains, including the variants of the type strain ATCC_23344T (JHU, GB 8 Horse, FMH) and the strains 2002721274, 2002721277, 2000031281 have been deposited with the United States as their geographical origin. Like the derivatives of the type strain (originally isolated in China from a soldier’s knee fluid), the strains 2002721274, 2002721277, 2000031281 grouped with strains from China. The strain 2000031281 was identical to the reference strain China7 (alias of ATCC23344T), the strain 2002721274 differed in 29 alleles from the strain China7. The strain 2002721277 belonged to the China5 group with 50 alleles difference from the strain China5 (**Fig 1A**). Another strain of the CDC (Ft. Detrick) strain collection, 2002721276, without given origin grouped between the China5 (CHN1) and China7 (CHN2) cluster. In summary, all of the strains that are on record with the United States as their geographic origin are originally from China, with the exception of BURK080, which originated in Turkey. The strain BURK081 was most closely related with only one allele difference to the strain A193, which was deposited as isolated in France in 1964. Since both strains differ in only one single allele, they can be regarded as a single strain. However, it remains uncertain whether the US or France is the correct origin of both varieties. Another possibility is that both strains do not come from the USA or France, as the closest related strain (2002721280) with 142 alleles difference comes from Iran.

The genomes of strains 2002734306, FDAARGOS_586 and L3_0746 have been deposited in the NCBI database with UK as their geographical origin and relate to the strain NCTC_120, also known as Lister strain or strain A, which was isolated in 1920 at the Lister Institute in London. The three strains grouped together with 0 to 1 allele difference and thus represent derivatives of NCTC_120. From cgMLST analysis it is unlikely that UK is actually the geographic origin as strain NCTC_120 is most closely related (160 alleles difference) with strain 6 (alias NCTC_10248) which was isolated in Turkey.

The genome sequence of strain 2000031065 was deposited in the database without historical records. In the Literature this strain is mentioned as strain Turkey_1 [36] (PLoS One. 2015; 10(9): e0137578). Indeed, this strain clustered most closely to strain Turkey_1 from Turkey but with a difference of 17 alleles (**Figs 1A and S2**). Another strain without record, SR092700I (alias BMP) was most closely related with two alleles difference to strain IVAN (NCTC_10229) from Hungary and can be regarded as a derivative of this strain.

Finally, the geographical origin of all the strains deposited from the Obolensk State Collection of Pathogenic Microorganisms (SCPM), Russia, remains uncertain. However, since they are descended from the V120 strain, a strain that was isolated from a horse in Russia in 1985, Russia actually appears to be the most likely origin. An exception was the strain SCPM-O-B-7093, which did not group with the Russian strains, but was most closely related to the strains L3_586 (Zagreb) and L3_580 (Mukteswar). Strain SCPM-O-B-4688 also did not group to the Russian cluster but showed 196 alleles difference to strain Turkey_9. In summary, the cgMLST analysis allowed the geographical allocation of strains with uncertain origin.

### Impact of sequencing technologies and assembly strategies

The presence of sequencing data from a given strain and its derivatives in the database, generated by different sequencing centers (e.g. USAMRIID, TIGR, Los Alamos) using different sequencing technologies (PacBio, Illumina, 454- and shotgun sequencing) allowed us to determine the influence of different sequencing technologies on the robustness of the developed cgMLST assay.

Genomes reconstructed exclusively on the basis of PacBio-Reads (e.g. B. mallei FMH, **Figs 1 and 2**) differed in only one single allele from genomes that were generated with Illumina sequencing reads alone or by means of hybrid assembly of Illumina, PacBio and 454 reads (e.g. *B*. *mallei* FMH_23344T) (**Figs 1** and **2**).

Interestingly, two genome sequences related to the type strain ATCC_23344T (ATCC_23344T: NC_006348.1, NC_006349.2 and JHU_2: AAIR00000000.1) that were sequenced in 2005 with the Shotgun Sanger Sequencing Technology [35] showed a higher number of different alleles (4, and 5, respectively) compared to genome sequences that were generated using next generation sequencing (**Fig 1A**, CH2, **Fig 2**).

For two strains (L3_0543, L3_552) the influence of different assembly strategies was assessed by testing different assemblers (Velvet, Spades, Skesa, CLC) implemented in SeqSphere and CLC Genomics Workbench software. It turned out that neither the sequencing technology nor the different assemblers had an influence on the results of the cgMLST analysis; no allelic differences were detected (**Fig 1A**). In addition, analysis of the raw sequencing data showed that *B*. *mallei* L3_0543 genomes obtained from Illumina reads compiled with and without Fast QC using SKESA were grouped with no allele difference. These findings underline the robustness of the developed cgMLST assay.

### Genetic variability after laboratory and host passage

Knowledge of strain diversification that occurs through multiple cultivation passages or through the host passage is of paramount importance in establishing strain identity.

Therefore, the definition of allelic distance cutoffs for the identification of outbreak complexes is an urgent need in next-generation sequencing (NGS) -based pathogen monitoring to enable accurate strain identification and source tracking in an outbreak scenario. Three key pieces of information are critical to defining and calibrating the cutoff: (1) information about the allele distances resulting from repeated cultivation and host passage of a given strain; (2) Availability of strains from precisely defined outbreak scenarios or strains with evidence of cultivation; (3) Knowledge of the influence of different sequencing approaches and assembly strategies to rule out sequencing and sequence analysis artifacts that could lead to misinterpretation of the results. The various genome sequences of the type strain ATCC_23344T and derivatives allowed us to address point (1). Point (2) could be assessed by analysis of outbreak strains from Dubai. Point (3) was estimated using information on sequencing technologies of the deposited genome sequences and by own analysis using various assembly strategies.

Based on this information, the cluster analysis carried out here showed that allelic differences of 1–3 alleles correspond to a single strain or close derivatives of the strain. The cutoff of 3 alleles was verified by applying the Pearson’s chi-square test with Yates’ continuity correction by comparing the probability of occurrence of allele differences of zero to three alleles in the single strain and derivative group with the single strain group. The x-square was found to be 40.34 and the p-value to be 2.12e-10, which actually shows that the distribution of allele differences from zero to three alleles was different in both groups.

## Discussion

Although glanders is considered a re-emerging disease in endemic countries, it is still rarely diagnosed because of missing diagnostic capacities. Only in a few cases the isolation of living *B*. *mallei* strains from diseased animals is successfully carried out and may require passage through an animal host. As a result, *B*. *mallei* strains and in particular their genome sequences are hardly accessible to the general public. A total of 83 genome sequences from 14 countries are currently available in the genome database. However, this figure is misleading as a significant number of *B*. *mallei* strains exist as duplicates and derivatives under different pseudonyms. After removing variants and duplicates from the database, 51 unique *B*. *mallei* genomes stored at the NCBI were identified. In addition, the majority of the genome sequences in the database belong to strains isolated many decades ago, while only a limited number of genomes are related to recently isolated *B*. *mallei* [37–39]. In the current study, 37 additional *B*. *mallei* genomes from the in-house strain collection were made available. These include 23 genomes of more recent outbreak strains, six *B*. *mallei* genomes from NCTC reference strains and four other undocumented strains as well as the genomes of the type strain ATCC 23344T, Mukteswar and Zagreb.

*B*. *mallei* is strictly clonal and the correct delineation of individual strains requires high-resolution typing assays. Although MLVA has proven to be suitable to differentiate individual *B*. *mallei* strains [14, 16, 17, 19], it requires profound laboratory skills and standardization in order to ensure robustness and reproducibility [20]. As a consequence, typing results obtained by MLVA may vary significantly between different laboratories when applied to an identical strain panel. Indeed, in addition to a high discriminatory power, robustness and repeatability are crucial parameters for molecular typing in order to ensure repeatable strain identification. The main aim of this study was therefore to provide a highly reproducible and easy-to-use core genome-based typing scheme for standardized high-resolution genotyping of *B*. *mallei* that facilitates epidemiological outbreak analysis and source tracking. To achieve this goal, we have developed a cgMLST assay consisting of more than 3,300 gene targets which allows high resolution typing of *B*. *mallei* with a resolution similar to MLVA and SNP analysis. The developed cgMLST assay was applied to a total of 120 *B*. *mallei* genomes, including 37 new genome sequences from strains of the in-house collections. By using Illumina sequencing and multiple raw read assembly strategies (Velvet, SKESA, Spades, and CLC) we could show that typing results are independent from assembly strategies. This underlines the excellent robustness and repeatability of the developed assay. Technical robustness could also be assessed by the analysis of twenty genomes of ten *B*. *mallei* strains from Turkey (Turkey 1 to Turkey 10 and Turkey 1_2 to Turkey 10_2). The genome sequences of these strains were sequenced and deposited by two different submitting institutions using different sequencing technologies (Illumina/454 and PacBio) and assembly strategies (newbler/velvet and HGAP). Despite the different technologies used, strains with the same designation differed in only 0 to 1 allele (**Fig 1A**).

In addition to the technical robustness and reproducibility of the assay, which are essential prerequisites for precise typing, knowledge of the genetic variation that occurs in the laboratory or in the host during an infection is essential. This knowledge is also important for assay calibration and the interpretation of typing results in connection with the question of attribution in forensic trace-back analyzes. In this study, we were able to show that a certain strain and its derivatives, which are available from different strain collections, show a difference of zero to two alleles in the cgMLST analysis, even when different assembly strategies are used for the genome construction. For example, genome sequences of the *B*. *mallei* type strain ATCC 23344T showed no allelic changes after passages in animals (horses) and humans (laboratory infection). Based on this finding, we assume that a difference of at least 3 alleles defines different strains. As a result, genome sequences that differ in more than three alleles are likely to represent different strains. In addition, this assumption is supported by the results of the cgMLST analysis of *B*. *mallei* strains, which are available in various strain collections and whose genomes are stored in the NCBI database. A given strain and derivatives of that strain, available in different strain collections, only showed a difference of 0 to two alleles. Applying this cutoff to the results of 23 outbreak strains, it can be concluded that the Dubai outbreak was caused by a single strain, while multiple strains were involved in the Bahrain outbreak. While Dubai strains were either indistinguishable or only differed in one allele, Bahrain strains were genetically more diverse (up to 16 alleles difference) and formed two different clusters (**Fig 1A and 1C**). This assumption is supported by the underlying different epidemiological backgrounds of the two outbreaks. In Dubai, the three horses affected by the outbreak were all imported from Syria at the same time and developed clinical symptoms of glanders during the quarantine within the quarantine facility. Hence, infection with a single *B*. *mallei* strain is very likely and is supported by the cgMLST data. In contrast, in Bahrain, the outbreak affected a larger area in the north and lasted two years (2010 to 2011). During the Bahrain outbreak, *B*. *mallei* was isolated from nine different horses imported from Syria and Kuwait and a local camel. Assuming that strains with more than three allele differences represent different strains, the outbreak in Bahrain was likely caused by five different but closely related *B*. *mallei* strains.

In terms of genetic resolution, cgMLST was equivalent or superior to the previous SNP genotyping [14]. While the SNP analysis could not differentiate between strains from Dubai ([14] **Fig 1B**), some strains showed one allele difference in cgMLST (**Fig 1B**). Although cgMLST only contains coding sequences for comparison purposes, the genetic resolution often resembles SNP-based analyses, as polymorphisms within intergenic regions are often excluded in SNP analysis due to poor coverage or genomic complexity [40, 41]. This applies in particular to *B*. *mallei*, where the associated genome is very variable and large-scale deletions are very common and therefore these regions cannot be used for the SNP analysis. As a result, the SNP assay used in the previous study to differentiate between *B*. *mallei* consisted of 242 SNPs, of which only 44 were variable between Dubai and Bahrain outbreak strains [14].

As reported for other pathogens [42–45], *B*. *mallei* showed in previous MLVA and SNP analysis spatial clustering [14–17, 39]. Spatial relationships could also be resolved using the newly developed cgMLST assay. In the cgMLST analysis, strains from different geographical regions, with no epidemiological link, differed clearly by at least 70 alleles, while strains with a known epidemiological link were separated by only a few alleles (**Fig 1A**). In some countries (Turkey, China) strains grouped in different clusters with 53 to 288 allelic differences (**S1 and S2 Figs**). This indicates the presence of different *B*. *mallei* lineages in these countries, as previously demonstrated by MLVA for strains from Pakistan [17]. Within a given cluster of India, allele differences of 0 to 22 were detected (**S3 Fig**, IND1), suggesting a common geographical origin. However, determining spatial relationships for *B*. *mallei* remains difficult as only a limited number of strains from each country are available.

In summary, the developed cgMLST assay offers an excellent combination of high genetic resolution and reproducibility for typing of *B*. *mallei*, resolving the population structure at bacterial strain level. The further application of the assay will allow us to gain more information on natural strain diversity and outbreak clusters, and will help to prevent false attribution of bacterial strain designations.

## Supporting information

S1 Fig Allelic profiles of *B*. *mallei* strains forming the Chinese clusters (CH1, CH2). The Minimum Spanning Tree was reconstructed on results provided by the cgMLST analysis. Each sequence type is represented by a single node, nodes are connected if they are single locus variants. Number along the branches indicate allelic differences. Nodes with less than 3 allelic differences to each other are shaded in grey. Each *B*. *mallei* strain is identified by a unique identifier. Spatial clustering of *B*. *mallei* strains dependent on the reported geographical origin.
(DOCX)

S2 Fig Allelic profiles of *B*. *mallei* strains forming the turkey clusters (THR1, THR2, THR3). The Minimum Spanning Tree was reconstructed on results provided by the cgMLST analysis. Each sequence type is represented by a single node, nodes are connected if they are single locus variants. Number along the branches indicate allelic differences. Nodes with less than 3 allelic differences to each other are shaded in grey. Each *B*. *mallei* strain is identified by a unique identifier. Spatial clustering of *B*. *mallei* strains dependent on the reported geographical origin.
(DOCX)

S3 Fig Allelic profiles of *B*. *mallei* strains forming the Indian clusters (IND1, IND2, IND3). The Minimum Spanning Tree was reconstructed on results provided by the cgMLST analysis. Each sequence type is represented by a single node, nodes are connected if they are single locus variants. Number along the branches indicate allelic differences. Nodes with less than 3 allelic differences to each other are shaded in grey. Each *B*. *mallei* strain is identified by a unique identifier. Spatial clustering of *B*. *mallei* strains dependent on the reported geographical origin.
(DOCX)

S4 Fig Neighbor-Joining tree visualizing the clustering of *B*. *mallei* strains. (DOCX)

S1 Table *B*. *mallei* genomes included into the query genome. For each *B*. *mallei* genome, the strain ID and the NCBI Accession numbers are provided.
(DOCX)

S2 Table Information on *B*. *mallei* genomes included in the analysis. All listed genomes are available in public databases.
(XLS)

S3 Table The cgMLST assay. (CSV)

S4 Table *B*. *mallei* strains found to not cluster in compliance to referred strain IDs/ designations of strains. For each *B*. *mallei* strain the following information are given: *B*. *mallei* strain ID, NCBI Accession(s), Alias, the expected and identified spatial attributions. As additional information, for the expected and identified spatial attributions, the IDs of neighbor joining strains are given. A visual representation is to be found in **Fig 1**.
(DOCX)

S5 Table Listing of *B*. *mallei* genomes that were identified to be deposited in databases with missing or unclear records on spatial attribution. For each *B*. *mallei* strain the following information are given: *B*. *mallei* strain ID, NCBI Accession(s), and the recorded as well as the identified spatial attributions. A visual representation is to be found in **Fig 1**.
(DOCX)

S1 Data (DOCX)
