# Supplementary material for: Genetic diversity and spatial distribution of Burkholderia mallei by core genome-based multilocus sequence typing analysis
Source: PLoS One. 2022 Jul 6;17(7):e0270499. doi: 10.1371/journal.pone.0270499 (PMC9258848; doi:10.1371/journal.pone.0270499)
Supplement: S2 Fig — The Minimum Spanning Tree was reconstructed on results provided by the cgMLST analysis. Each sequence type is represented by a single node, nodes are connected if they are single locus variants. Number along the branches indicate allelic differences. Nodes with less than 3 allelic differences to each other are shaded in grey. Each B. mallei strain is identified by a unique identifier. Spatial clustering of B. mallei strains dependent on the reported geographical origin. (DOCX) [file pone.0270499.s002.docx]

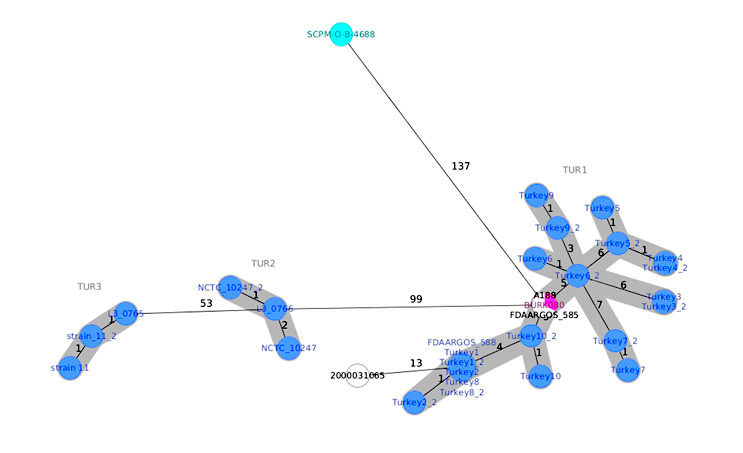

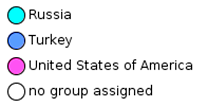


**S2 Fig.** Direct cgMLST comparison of *B. mallei* strains forming the Turkey clusters (THR1, THR2, THR3). The Minimum Spanning Tree was reconstructed on results provided by the cgMLST analysis. Spatial clustering of *B. mallei* strains dependent on the reported geographical origin as shown in the legend. Each sequence type is represented by a single node, nodes are connected if they are single locus variants. Number along the branches indicate allelic differences. Nodes with less than 3 allelic differences to each other are shaded in grey. Each *B. mallei* strain is identified by a unique identifier. Spatial clustering of *B. mallei* strains dependent on the reported geographical origin.
