# Supplementary material for: Genetic diversity and spatial distribution of Burkholderia mallei by core genome-based multilocus sequence typing analysis
Source: PLoS One. 2022 Jul 6;17(7):e0270499. doi: 10.1371/journal.pone.0270499 (PMC9258848; doi:10.1371/journal.pone.0270499)
Supplement: S4 Fig — (DOCX) [file pone.0270499.s004.docx]

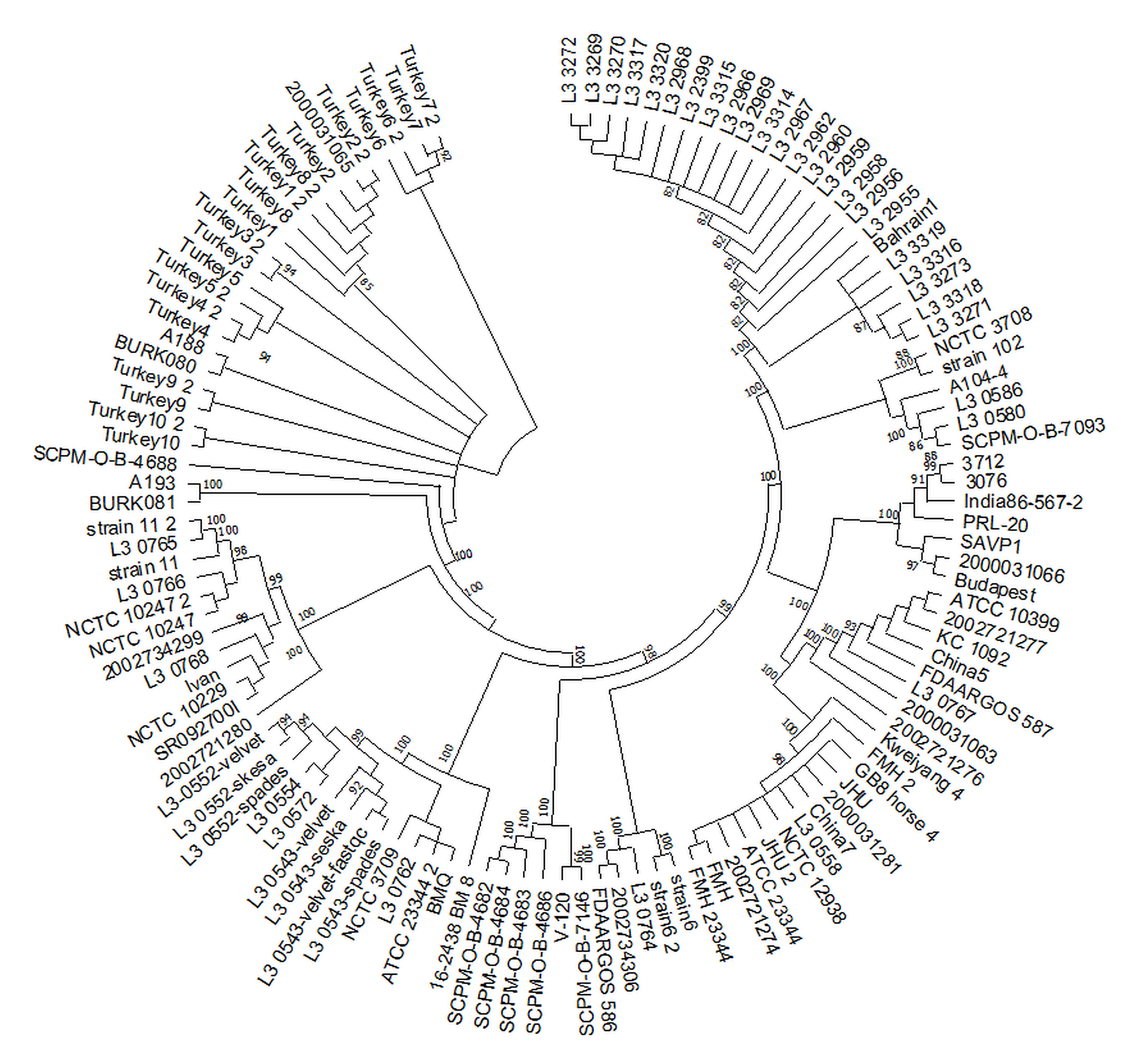


**S4 Fig.** Neighbor-Joining Tree visualizing the clustering of *B. mallei* strains. The bootstrap consensus tree inferred from 500 replicates. Bootstraps below 80 were removed.
