# Supplementary material for: Genetic diversity and spatial distribution of Burkholderia mallei by core genome-based multilocus sequence typing analysis
Source: PLoS One. 2022 Jul 6;17(7):e0270499. doi: 10.1371/journal.pone.0270499 (PMC9258848; doi:10.1371/journal.pone.0270499)
Supplement: S1 Table — For each B. mallei genome, the strain ID and the NCBI Accession numbers are provided. (DOCX) [file pone.0270499.s005.docx]

**S1 Table.** *B. mallei* genomes included into the query genome. For each *B. mallei* genome, the strain ID and the NCBI Accession numbers are provided.

| *B. mallei* strain ID | NCBI Accessions |
| --- | --- |
| strain 11 | NZ_CP009587.1, NZ_CP009588.1 |
| 2000031063 | NZ_CP008732.1, NZ_CP008731.1 |
| 2002721276 | NZ_CP010065.1, NZ_CP010066.1 |
| 2002734299 isolate SR092700C | NZ_CP009337.1, NZ_CP009338.1 |
| Bahrain1 | NZ_CP017175.1, NZ_CP017176.1 |
| India86-567-2 | NZ_CP009642.1, NZ_CP009643.1 |
| NCTC 10247 | NZ_CP007802.1, NZ_CP007801.1 |
| SAVP1 | NC_008785.1, NC_008784.1 |
| NCTC 10229 | NC_008836.1, NC_008835.1 |
| strain 6 | NZ_CP008711.1, NZ_CP008710.1 |
| BMQ | NZ_CP008723.1, NZ_CP008722.1 |
| 2002734306 | NZ_CP009707.1, NZ_CP009708.1 |
| Turkey2 | NZ_CP009727.1, NZ_CP009728.1 |
