# Supplementary material for: Genetic diversity and spatial distribution of Burkholderia mallei by core genome-based multilocus sequence typing analysis
Source: PLoS One. 2022 Jul 6;17(7):e0270499. doi: 10.1371/journal.pone.0270499 (PMC9258848; doi:10.1371/journal.pone.0270499)
Supplement: S4 Table — For each B. mallei strain the following information are given: B. mallei strain ID, NCBI Accession(s), Alias, the expected and identified spatial attributions. As additional information, for the expected and identified spatial attributions, the IDs of neighbor joining strains are given. A visual representation is to be found in Fig 1. (DOCX) [file pone.0270499.s008.docx]

**S4 Table.** *B. mallei* strains found to not cluster in compliance to referred strain IDs/ designations of strains. For each *B. mallei* strain the following information are given: *B. mallei* strain ID, NCBI Accession(s), Alias, the expected and identified spatial attributions. As additional information, for the expected and identified spatial attributions, the IDs of neighbor joining strains are given. A visual representation is to be found in **Fig 1**.

| *B. mallei* strain ID | NCBI Accession(s) | Alias | Expected spatial attribution (neighbor joining stains) | Identified spatial attribution (neighbor joining stains) |
| --- | --- | --- | --- | --- |
| ATCC 23344_2 | NZ_CP008704.1, NZ_CP008705.1 | NBL 7, 3873, EY 2233, RH 627, China7, 000031304, GB8, GB15.1-2, NCTC 12938, FDAARGOS_590 | China (strains: FMH, NCTC 12938) | Turkey (strains: BMY, NCTC 3709) |
| FDAARGOS 587 | NZ_RKJX00000000.1 | ATCC 15310, NCTC 10230, 2000031063, GB12, Ivan | Hungary (NCTC 10299, Ivan) | China (strains: China5, ATCC 10399) |
| 2000031063 | NZ_CP008732.1, NZ_CP008731.1 | ATCC 15310, NCTC 10230, FDAARGOS 587, GB12, Ivan | Hungary (NCTC 10230, Ivan) | China (strains: China5, ATCC 10399) |
| KC_1092 | NZ_CP009942.1, NZ_CP009943.1 | Pasteur Inst 53-236, 2002721280 | Iran (strain: CDC 2002721280; Turkey strains in the Turkey group) | China (strains: 2000031063, L3_0767) |
| Budapest | LUFQ00000000.1 | NCTC 10229 | China (strain: NCTC 10299) | India (strains: Ivan, 2002734299) |
