# Supplementary material for: Genetic diversity and spatial distribution of Burkholderia mallei by core genome-based multilocus sequence typing analysis
Source: PLoS One. 2022 Jul 6;17(7):e0270499. doi: 10.1371/journal.pone.0270499 (PMC9258848; doi:10.1371/journal.pone.0270499)
Supplement: S5 Table — For each B. mallei strain the following information are given: B. mallei strain ID, NCBI Accession(s), and the recorded as well as the identified spatial attributions. A visual representation is to be found in Fig 1. (DOCX) [file pone.0270499.s009.docx]

**S5 Table.** Listing of *B. mallei* genomes that were identified to be deposited in databases with missing or unclear records on spatial attribution. For each *B. mallei* strain the following information are given: *B. mallei* strain ID, NCBI Accession(s), and the recorded as well as the identified spatial attributions. A visual representation is to be found in **Fig 1**.

| *B. mallei* strain ID | NCBI Accession(s)/ Genome Accession | Recorded spatial attribution (country) | Identified  spatial attribution  (country) |
| --- | --- | --- | --- |
| BURK080 | LUFN01 | United States | Turkey |
| BURK081 | LUFM01 | United States | Remains unclear, France |
| 2002734306 | NZ_CP009707.1, NZ_CP009708.1 | United Kingdom | None (next to a Russian and Turkey strains) |
| FDAARGOS_588 | NZ_CM012068.1 | unknown | Turkey |
| FDAARGOS_589 | NZ_CM012066.1 | unknown | India |
| 2000031065 | JXNW01 | unknown | Turkey |
| 2002721276 | NZ_CP010065.1, NZ_CP010066.1 | unknown | China |
| 2002721274 | JXQN01 | United States | China |
| 2002721277 | JXNX01 | United States | China |
| 2000031281 | AWGQ01 | United States | China |
| SCPM-O-B-7093 | RBMI01 | Unknown | Indonesia |
| L3_0552 | provided in Table 1 | unknown | India |
| L3_0554 | provided in Table 1 | unknown | India |
| L3_0543 | provided in Table 1 | unknown | India |
| L3_0572 | provided in Table 1 | unknown | India |
