# Supplementary material for: Genetic diversity and spatial distribution of Burkholderia mallei by core genome-based multilocus sequence typing analysis
Source: PLoS One. 2022 Jul 6;17(7):e0270499. doi: 10.1371/journal.pone.0270499 (PMC9258848; doi:10.1371/journal.pone.0270499)
Supplement: S1 Data — (DOCX) [file pone.0270499.s010.docx]

**S1 Data**

**1 Strains not clustering according to their designation**

One genome sequence deposited as ATCC 23344_2 (NZ_CP008704.1, NZ_CP008705.1) did not cluster with the type strain group (**Figures 2**) but clustered with only one allele difference next to *B. mallei* strain NCTC 3709 which belongs to a separate phylogenetic lineage with more than 180 alleles difference to the type strain group.

Strain KC_1092 (SAMN03198318) is an alias of strain CDC 200272180 isolated in Iran. However, the strain deposited as CDC 200272180 (SAMN02435874) did not cluster with KC_1092 but correctly with strains of a similar geographic origin (Turkey). Altogether, the cluster around Chinese strains China5 and ATCC 10399 contained three strains of doubtful affiliation (KC_1092, FDAARGOS 587 and 2000031063). The fifth remaining strain that did not cluster as expected was strain BUDAPEST (SAMN04260157) which should be identical to NCTC 10229. However, strain “BUDAPEST” did not match NCTC 10229 (SAMN02604032) but instead clustered with strains SVAP1, 3076 and India86-567-2, all isolated in India.
